# Systematis Nosologici Novi, Exemplum Parvulum

**Published:** 1822-04

**Authors:** Roberto Tytler

**Affiliations:** A.D. M,DCCC,XXI.


THE LONDON

### Medical and Physical Journal.

4 OF VOL. XLVII.] APRIL, 1822. [No. 278.

For many fortunate discoveries in medicine, and for the detection of numerous errors, the world is indebted to the rapid circulation of Monthly Journals; and there never existed any work, to which the Faculty, in Europe and America, were under deeper obligations, than to the Medical and Physical Journal of London, now forming a long, but an invaluable, series.—RUSH.

### Original Communications, Select Observations, etc.

### NOSOLOGY.

*To the Editor of the Medical and Physical Journal.*

SIR,

THE accompanying specimen of a new system of Nosology, I have just received from a very intelligent medical friend in India, Dr. ADAM, assistant physician to the General Hospital at Calcutta. As it is interesting, from its novelty,—from the peculiar views which it contains,—and from the place where it is published, I think it may prove acceptable to the profession in England: you will, therefore, oblige me by giving it an early insertion in your widely-circulated and very respectable Journal. I am, &c.

JOHN WEBSTER, M.D.

&c. &c.

*3, Little George-street, Westminster;*

*6th March,* 1822.

ART. I.—*Systematis Nosologici Novi, Exemplum Parvulum.* Auctore ROBERTO TYTLER, M.D.—A. D. M,DCCC,XXI.

LECTORI ÆQUO.

POST tempestates pluviales, scilicet in mensibus autumnalibus, semina Oryzæ, quæ Indice *Ouse*, *Ballum*, *Patcherry*, *Moongy*, *Sattee*, et *Rarha* vocantur, et Anglice *Coarse Rice*, *Cargo*, et *Yellow Patna* nominantur, tumefacta sunt, colorem nigrum, rubrum, vel flavum exhibent, et fætorem putridum exhalent. Super grani utriusque superficiem, tunica densa, crassa, oleaginosa, venenifera, quam Indi *Koora* et *Kun* vocant, extendens visa est; quamobrem animalia, ex illius Oryzæ usu, maxime ægrotant. Frequenter aliorum frumentorum semina, scilicet Tritici et Secalis, a pluviis multis vitiosa fiunt; sed Oryza quotannis depravata fit, cujus quantitates immensæ pro cibo hominum atque animalium ad mercatorum fora introductæ sunt. De illis causis morborum oriundorum, quædam genera et quasdam species hoc tentamen triviale haud valde explicat.

### TABULA PRIMA, CLASSEM EXHIBENS.

CLASSIS. *Morbi Cereales.*

Actio morbida excitata a seminibus frumentorum vitiosis in ventriculum admissis; debilitate pyrexiam consequente; quorumdam organorum inflammatio, etiam gangrena.

| *Genera.* | *Species.* |
|---|---|
| Tritico vitioso. | Typhus mitior. |
| | Typhus gravior. |
| | Sudor Anglicanus, vel Ephemera maligna Sudatoria. |
| Morbus Triticeus. | Variola Triticea gravior. |
| | Variola Triticea mitior. |
| | Varicella. |
| Secali vitioso. | Ignis Sacer. |
| | Ignis Sancti Antonii. |
| Morbus Secaleus. | Cholera Secalea. |
| | Pestis Secalea. |
| | Variola Secalea. |
| | Gangrena Sicca. |
| Oryza vitiosa. | Febris remittens. |
| | Typhus icterodes. |
| Morbus Oryzeus. | Cholera Oryzea, vel Mort de Chien, vel Cholera spasmodica. |
| | Pestis Oryzea. |
| | Variola Oryzea. |
| | Dysenteria. |
| | Cachexia Oryzea, vel Beri-Beri. |

### TABULA SECUNDA, MORBI TRITICEI GENUS ET QUASDAM SPECIES EXHIBENS.

GENUS—*Morbus Triticeus.*

Actio morbida excitata ab Tritico depravato in ventriculum admisso; debilitate pyrexiam consequente; dolor abdominalis; inflammatio visceralis; sæpe gangrena et exanthema, sive super cutem eruptio.

SPECIES I^ma^.—*Typhus Mitior.*

Anxietate, lassitudine, et rigore pyrexiam præcedentibus; inflammatio erythematica mitior ventriculi, intestinorumque; dolor abdominalis; vomitus; vertigo; communis sensorii affectus; lingua alba, humida, nonnunquam arida; corpus minime calidum; pulsus numeris auctis non vigore; sitis; singultus; facies Hippocratica; mors.

Species II<sup>da</sup>.—*Typhus Gravior.*

Horrore pyrexiam præcedente; inflammatio erythematica gravior ventriculi; gangrena intestinorum, etiam cutis, sive lividæ maculæ petechiales sub cuticulâ erumpentes; totius corporis dolor; sensorium multum affectum; vomitus; dejectio frequens; initio calor auctus; pulsus vehemens, decrescens; sitis maxima; lingua arida et nigra; halitus fætor; singultus; mors.

Species III<sup>tia</sup>.—*Sudor Anglicanus, vel Ephemera maligna Sudatoria.*

Instanter debilitate maximâ pyrexiam consequente; sudor multus; gangrena ventriculi, etiam intestinorum; mors.

Species IV<sup>ta</sup>.—*Variola Triticea gravior.*

Pyrexiam apud initium mitem, tum valde vehementem, rigores comitantes; dolor abdominis; vertigo; sensorii communis affectus; inflammatio gravior erythematica ventriculi et intestinorum; eruptio aquosa, maligna, confluens, super cutem extendens; lingua arida et nigra; halitus fætor; sitis; pulsus diminutus; singultus; mors.

Species V<sup>ta</sup>.—*Variola Triticea mitior.*

Rigore pyrexiam haud vehementem præcedente; calor auctus; pulsus frequens; inflammatio mitior visceralis erythematica; dolor abdominalis; vertigo; eruptio granulosa super cutem distincta et purulenta; sitis; lingua arida; nonnunquam mors.

Species VI<sup>ta</sup>.—*Varicella.*

Pyrexia minima; inflammatio intestinalis nulla; eruptio super cutem mitis, aquosa, tum purulenta et distincta; lingua humida, alba.

TABULA TERTIA, MORBI SECALEI GENUS ET QUASDAM SPECIES EXHIBENS.

Genus—*Morbus Secaleus.*

Actio morbida excitata ab Secalia depravato in ventriculum admisso; pyrexiam debilitate maximâ frequenter consequente; inflammatio visceralis gravior; sæpius gangrena organorum.

Species I<sup>ma</sup>.—*Ignis Sacer.*

Plantarum et palmarum cutis ardens; pyrexiam horrore præcedente; dolor abdominalis acer; vomitus; vertigo; canalis intestinalis gangrena; trunci et artuum musculorum spasmi acuti et vehementes; mors.

Species II<sup>da</sup>.—*Ignis Sancti Antonii.*

Totius cutis ardor et formicatio; pyrexia maxima; gangrena visceralis; spasmi vehementes; mors.

Species III$^{tia}$.—*Cholera Secalea.*

Apud initium rigore pyrexiam præcedente, tum corporis calore aucto, postea corpore instanter frigescente, et debilitate maximâ consequente; inflammatio erythematica ventriculi, intestinorumque, dolor abdominalis acer; vomitus, atque dejectiones aquosæ frequentes; vertigo; spasmi musculorum acuti; pulsus diminutus; sudores frigidi; mors.

Species IV$^{ta}$.—*Pestis Secalea.*

Horrore pyrexiam præcedente; inflammatio erythematica gravissima, et gangrena ventriculi intestinorumque; halitus fætor; lingua arida; vertigo; vomitus; glandularum lymphaticarum tumores; mors.

Species V$^{ta}$.—*Variola Secalea.*

Indiciis simulantibus Variolam Triticeam graviorem.

Species VI$^{ta}$.—*Gangrena Sicca.*

Pyrexia; corporis extremitatum partium inflammatio, postea gangrena; mors.

TABULA QUARTA, MORBI ORYZEI GENUS ET QUASDAM SPECIES EXHIBENS.

Genus—*Morbus Oryzeus.*

Actio morbida excitata ab Oryza depravata in ventriculum admissa; pyrexiam sæpius debilitate maximâ consequente; inflammatio visceralis; nonnunquam gangrena organorum.

Species I$^{ma}$.—*Febris Remittens.*

Pyrexiam rigore præcedente; inflammatio erythematica ventriculi et intestinorum mitior; dolor abdominalis; calor auctus; pulsus frequens; vomitus; vertigo; communis sensorii affectus; sitis; singultus; mors.

Species II$^{da}$.—*Typhus Icterodes.*

Horrore pyrexiam præcedente; inflammatio erythematica gravior ventriculi; gangrena intestinorum; suffusio biliosa sub cuticulâ extendens; sensorii communis multus affectus; pulsus frequens, nonnunquam durus, tum instanter mollis; calor auctus, sæpe diminutus; vomitus nigri, biliosi; dejectiones nigræ; sitis; lingua arida; halitus fætor; singultus; mors.

Species III$^{tia}$.—*Cholera Oryzea.*

Apud initium, rigore et facie Oryzea pyrexiam præcedentibus, calore corporis aucto, tum instanter diminuto, et maximâ debilitate consequente; pulsus nullus; inflammatio ventriculi et intestinorum erythematica stellata; nonnunquam gangrena organorum; spasmi musculorum artuum, truncique, vehementiores, acuti; vertigo; vomitus et dejectiones aquosæ frequentes; sudores frigidi; mors.

Species IV$^{ta}$.—***Pestis Oryzea.***

Indiciis simulantibus Pestem Secaleam.

Species V$^{ta}$.—***Variola Oryzea.***

Indiciis simulantibus variolam Triticeam graviorem.

Species VI$^{ta}$.—***Dysenteria.***

Pyrexiam rigoribus comitantibus; coloni, rectique inflammatio, nonnunquam ulceratio et gangrena; dejectiones frequentes mucosæ; tenesmus; pulsus decrescens; corpore paulatim emacrescente; facies Hippocratica; mors.

Species VII$^{tima}$.—***Cachexia Oryzea.***

Corpore paulatim emacrescente; diarrhœa; abdominis et artuum tumores aquosi; nonnunquam organorum gangrena; dyspnœa; mors.

